# Supplementary material for: Temporal perturbations cause movement-context independent but modality specific sensorimotor adaptation
Source: J Vis. 2022 Feb 24;22(2):18. doi: 10.1167/jov.22.2.18 (PMC8883149; doi:10.1167/jov.22.2.18)
Supplement: Supplement 1 [file jovi-22-2-18_s001.pdf]

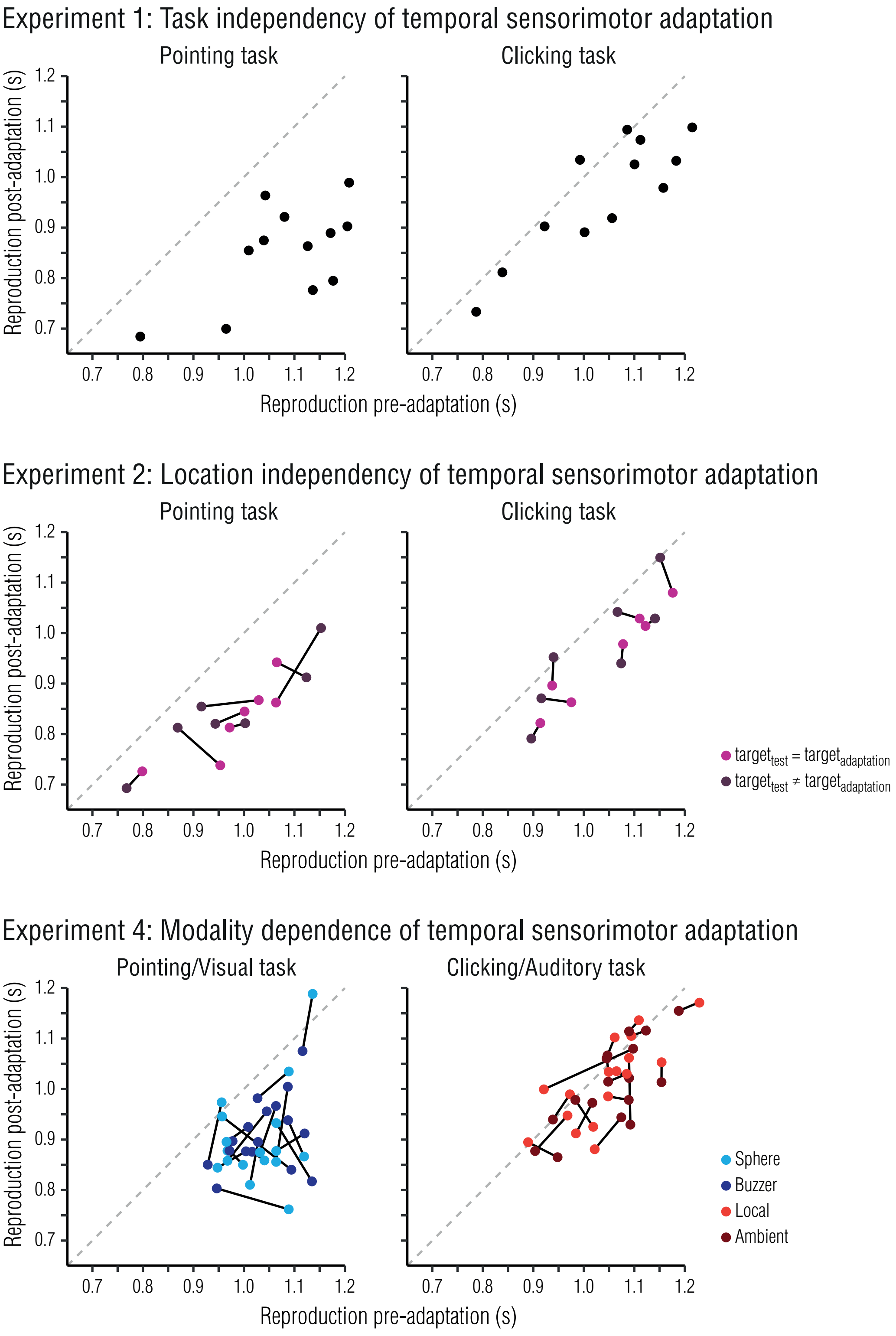

Figure S1. Single subject performance in Experiment 1, 2, and 4. Connected dots represent one subject in the different task conditions. Plotted as post-adaptation reproductions against pre-adaptation reproductions. Dots below the dashed line reflect effects of adaptation (under-reproduction in post-adaptation trials compared to pre-adaptation trials), and the distance to the dashed line reflects the strength of adaptation (larger distance - larger adaptation effect).
