# Supplementary material for: Temporal perturbations cause movement-context independent but modality specific sensorimotor adaptation
Source: J Vis. 2022 Feb 24;22(2):18. doi: 10.1167/jov.22.2.18 (PMC8883149; doi:10.1167/jov.22.2.18)
Supplement: Supplement 2 [file jovi-22-2-18_s002.pdf]

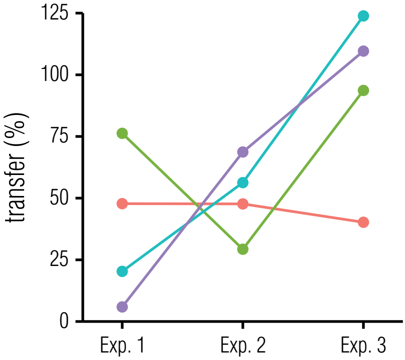

Figure S2. Within-subject performance for different task contexts. Transfer of adaptation for four participants who completed each Experiment (1-3). Connected dots represent one participant, additionally color-coded. Dots represent the transfer from pointing adaptation to the clicking task (Exp. 1), to the mirrored clicking task (Experiment 2), and to the non-VR pointing task (Experiment 3).
