# Supplementary material for: Temporal perturbations cause movement-context independent but modality specific sensorimotor adaptation
Source: J Vis. 2022 Feb 24;22(2):18. doi: 10.1167/jov.22.2.18 (PMC8883149; doi:10.1167/jov.22.2.18)
Supplement: Supplement 3 [file jovi-22-2-18_s003.pdf]

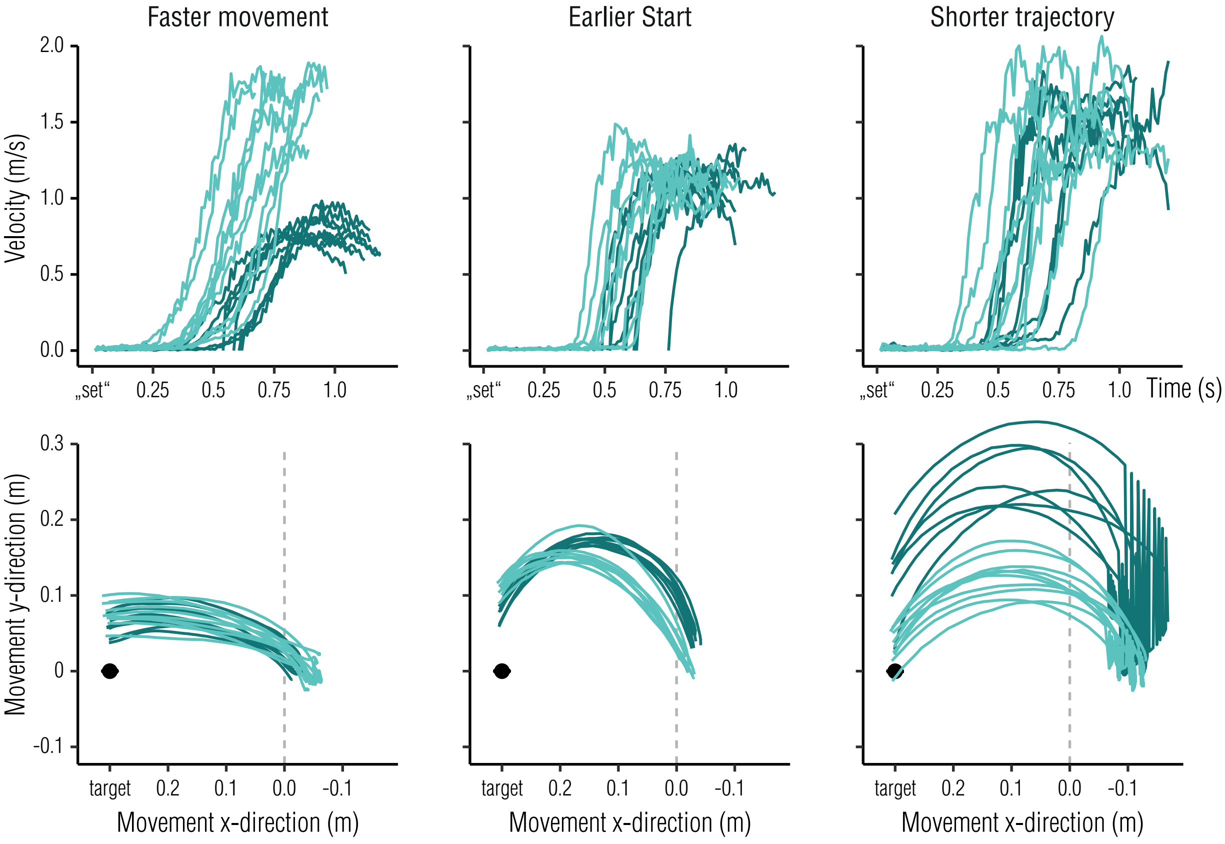

Figure S3. Movement profiles for three sample participants. Data depicted are single trial pointing test-trials, color-coded for pre- and post-adaptation trials. Top row shows velocity over time, starting from the "set" signal; the end of lines corresponds to reproduced durations. Bottom row shows trajectories, where the black dot represents the target, the dashed line represents the visual aid line that appears in the VR-environment. Pointing movements were performed from right to left. Columns represent three participants who adopted different strategies in response to the adaptation: performing faster movements (left top panel), starting the movement earlier (middle top panel), or shortening the trajectory (bottom right panel).
