# Supplementary material for: Temporal perturbations cause movement-context independent but modality specific sensorimotor adaptation
Source: J Vis. 2022 Feb 24;22(2):18. doi: 10.1167/jov.22.2.18 (PMC8883149; doi:10.1167/jov.22.2.18)
Supplement: Supplement 4 [file jovi-22-2-18_s004.pdf]

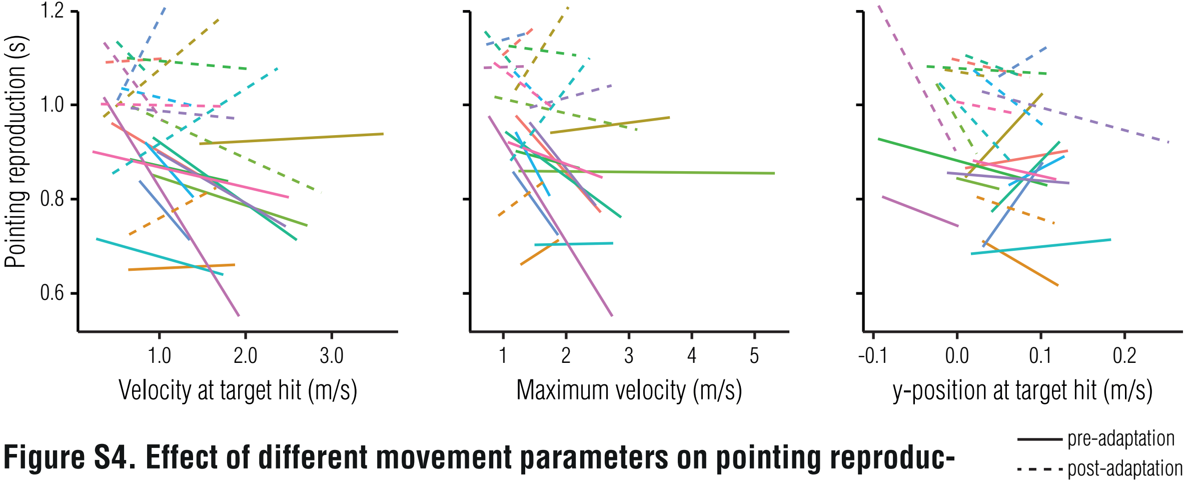

Figure S4. Effect of different movement parameters on pointing reproductions (Experiment 1). From left to right: Pointing reproductions as a function of velocity at target hit, maximum velocity throughout the pointing movement, and y-position at target hit. Lines represent linear fits for single subjects, separately for pre- and post-adaptation trials.
