# Supplementary material for: Temporal perturbations cause movement-context independent but modality specific sensorimotor adaptation
Source: J Vis. 2022 Feb 24;22(2):18. doi: 10.1167/jov.22.2.18 (PMC8883149; doi:10.1167/jov.22.2.18)
Supplement: Supplement 5 [file jovi-22-2-18_s005.docx]

**Supplementary Materials**

Nadine Schlichting, Tatiana Kartashova, Michael Wiesing, Eckart Zimmermann

**Details on the VR-environment for Experiment 1-3**

All experiments were conducted on a Windows 10 based desktop computer (Alienware Aurora R8, Intel(R) Core™ i7-8700 CPU @ 3.20 GHz, 16 GB RAM, NVIDIA GeForce GTX 1080TI graphics card) connected to an HTC Vive Pro Eye Head Mounted Display (HMD) (HTC Corporation, Taoyuan, Taiwan). The HMD presents stimuli on two low-persistence organic light-emitting diode (OLED) displays with a resolution of 1,440 x 1,600 pixels per eye and a refresh rate of 90 Hz. Additionally, participants used a Vive motion-controller for their right hand. The virtual environment (VE) was rendered using SteamVR and a custom-made program created in Unity game engine, version 2019.1.13f1 (Unity Technologies, San Francisco, U.S.). Head and hand movements were tracked via the HMD and controller using the SteamVR 1.0 tracking system. According to previous research, this system provides a robust tracking of head and hand motion with a 360° coverage, provided tracking loss is prevented (Niehorster et al., 2017). Tests of Verdelet et al. (2019) demonstrated a submillimeter precision (0.237 mm) and an accuracy of 8.7 mm for static and 8.5 mm for dynamic objects. While the system can update the user’s pose (position and orientation) at a higher rate (up to 1000 Hz for the HMD and 250 Hz the controllers), in this study the sampling rate for both HMD and controller was limited by the HMD’s refresh rate of 90 Hz. Because participants always responded to stimuli in front of them, we did not need the full coverage around participants in the present study. Hence, in order to minimize the chance of occlusions of the HMD or the controller and thereby avoiding tracking loss, our setup had both base stations facing the participant. Throughout the experiment, participants held the controller with an outstretched index finger placed on top of the controller with the fingertip matching the tracking origin of the controller as close as possible (see Figure 1A). Participants’ hands were presented as gloves instead of bare hands. Previous research has shown that the appearance of self-avatars can influence the self-perception and behavior of participants. Most notably the so-called Proteus Effect, which describes the tendency of participants to infer their expected behaviors and attitudes from their self-avatar’s appearance (Yee et al., 2009; Yee & Bailenson, 2007). By presenting gloves we were able to cover several characteristics that might otherwise be incongruent to the participants real hands, such as the skin color or gender of the hands.

**Details on the VR-environment for Experiment 4**

In contrast to previous experiments, Experiment 4 was created in Unreal Engine 4.25 (UE) and a new VE was developed for the experiment (Figure 5A). The VE consisted of a customized version of UE’s “Archviz Interior” sample project (EpicGames, 2019). The original sample makes use of real-time raytracing, a modern rendering technique that is currently too performance-intensive to meet the high performance demands of VR rendering. Instead, we replaced all ray-tracing effects by precalculated lighting and reflections. Additionally, all 3D models and textures were optimized (i.e., lowered in resolution) until we were able to hit stable 90 frames per second (FPS).

**References**

EpicGames. (2019). *New Archviz Interior Rendering sample project now available!* Unreal Engine. https://www.unrealengine.com/en-US/blog/new-archviz-interior-rendering-sample-project-now-available

Niehorster, D. C., Li, L., & Lappe, M. (2017). The accuracy and precision of position and orientation tracking in the HTC Vive Virtual Reality system for scientific research. *I-Perception*, *8*(3), 204166951770820. https://doi.org/10.1177/2041669517708205

Verdelet, G., Salemme, R., Desoche, C., Volland, F., Farne, A., Coudert, A., Hermann, R., Truy, E., Gaveau, V., & Pavani, F. (2019). Assessing spatial and temporal reliability of the Vive System as a tool for naturalistic behavioural research. *2019 International Conference on 3D Immersion (IC3D)*, 1–8. https://doi.org/10.1109/IC3D48390.2019.8975994

Yee, N., & Bailenson, J. (2007). The Proteus Effect: The Effect of Transformed Self-Representation on Behavior. *Human Communication Research*, *33*(3), 271–290. https://doi.org/10.1111/j.1468-2958.2007.00299.x

Yee, N., Bailenson, J. N., & Ducheneaut, N. (2009). The Proteus Effect: Implications of Transformed Digital Self-Representation on Online and Offline Behavior. *Communication Research*, *36*(2), 285–312. https://doi.org/10.1177/0093650208330254
